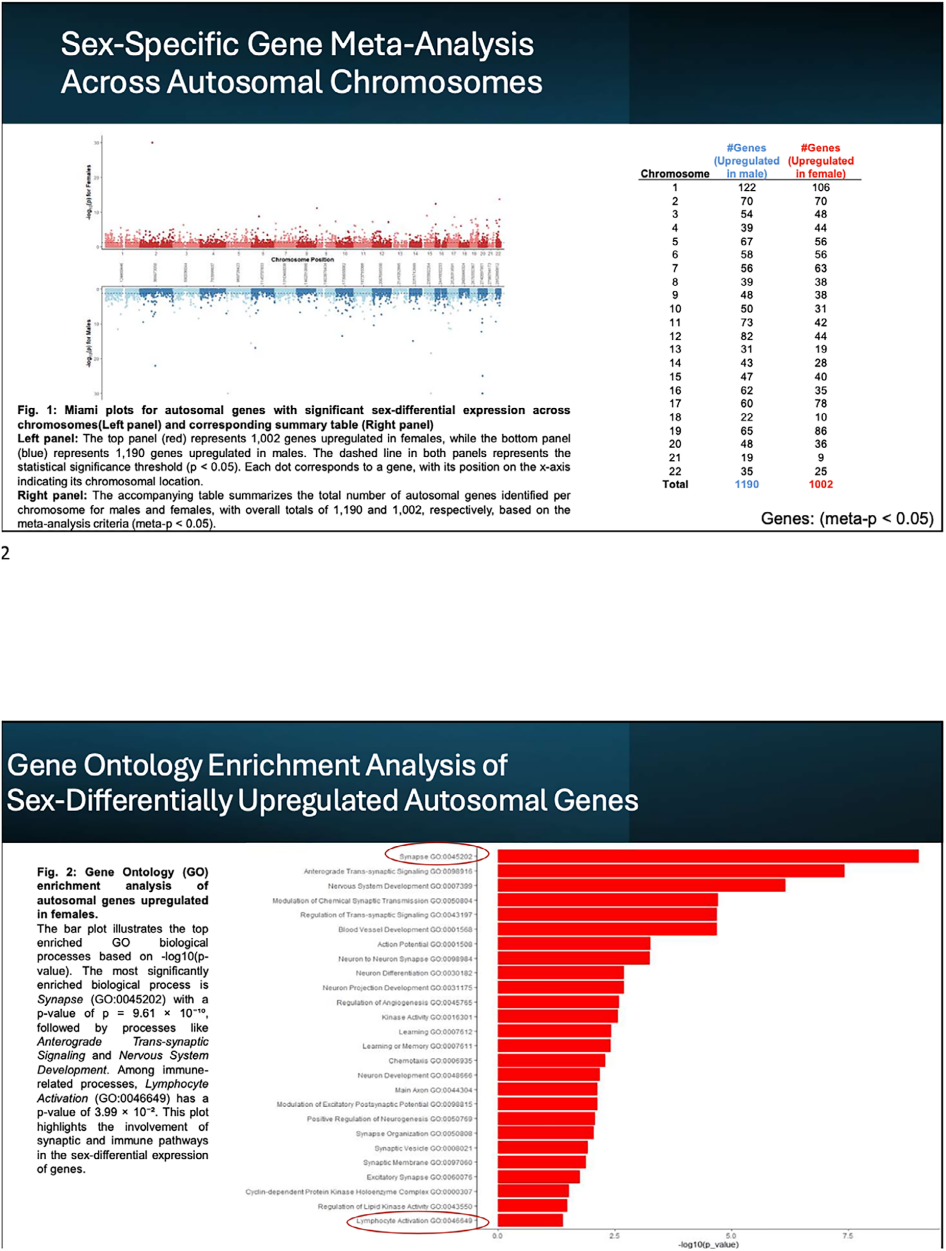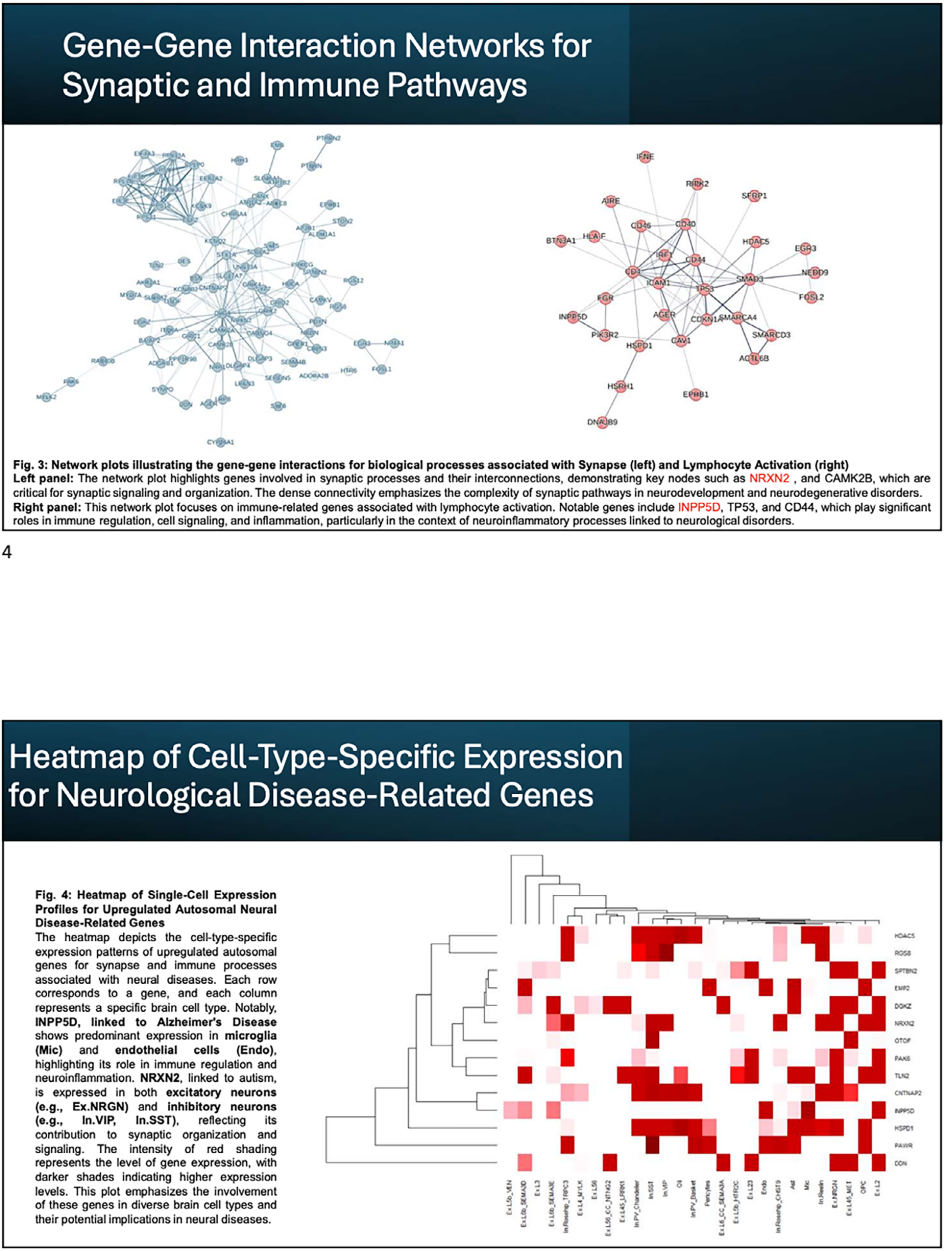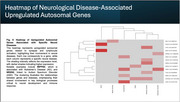# Brain Transcriptome Analysis Identifies Sex‐Associated Synaptic and Immune‐Associated Genes Involved in Alzheimer's Disease and Psychiatric Disorders

**DOI:** 10.1002/alz70855_097126

**Published:** 2025-12-23

**Authors:** Muyang Zhang, Xiaopu Zhou

**Affiliations:** ^1^ University of Toronto, Toronto, ON, Canada; ^2^ The Hospital for Sick Children, Toronto, ON, Canada

## Abstract

**Background:**

Sex differences significantly influence the risk and progression of neurodevelopmental and neurodegenerative disorders. Females exhibit higher susceptibility to Alzheimer's disease (AD), while males are more prone to Parkinson's disease and autism spectrum disorder (ASD). Despite these observations, the molecular mechanisms driving these sex differences remain poorly understood.

**Method:**

RNA‐seq data from 2,211 samples spanning 13 brain regions and 980 individuals were obtained from the GTEx database. Autosomal genes with sex‐differential expression were identified using robust linear regression, followed by meta‐analysis. Genes meeting the significance thresholds (*p* < 0.05 and Q_pval < 0.05) were subjected to Gene Ontology analysis to explore their biological functions. Associations with neurological disorders were assessed using annotations from the GWAS Catalog. Single‐cell RNA‐seq data from the PsychENCODE Knowledge Portal (syn25922167) were analyzed to investigate the cell‐type‐specific expression patterns of the identified genes.

**Result:**

A total of 2,294 genes met the significance criteria (*p* < 0.05 and Q_pval < 0.05). Among autosomal genes with sex‐differential expression, 1002 were more abundant in females and were primarily associated with neural‐related pathways (e.g., synapse; *p* = 9.61 × 10⁻¹⁰) and immune‐related pathways (e.g., lymphocyte activation; *p* = 3.99 × 10⁻²). Several of these neural and immune‐associated genes are linked to several psychiatric disorders and AD. For instance, synaptic genes such as *NRXN2* and *CNTNAP2* are associated with ASD, while *INPP5D*, an AD GWAS hit involved in lymphocyte activation, stands out. Single‐cell RNA‐seq analysis revealed cell‐type‐specific expression patterns of these sex‐associated genes. For example, INPP5D is predominantly expressed in microglia and endothelial cells, whereas NRXN2 is expressed in both excitatory and inhibitory neurons.

**Conclusion:**

This study highlights the critical role of sex‐specific gene expression in brain disorders, particularly psychiatric disorders and AD. Synaptic and immune‐associated pathways, mediated by key sex‐associated genes such as *NRXN2* and *INPP5D*, emerge as potential modulators of neurological disorders. These findings deepen our understanding of sex‐specific brain pathophysiology and offer a foundation for developing personalized therapeutic strategies targeting these mechanisms.